# Associations of COVID-19 lockdown with birth weight in China

**DOI:** 10.3389/fped.2024.1336108

**Published:** 2024-01-22

**Authors:** Yumeng Chen, Jingjie Fan, Xiaowei Li, Yufeng Ye, Yanyun Lv, Suijin Zheng, Jianxiong Hu, Yudong Pu, Tao Liu

**Affiliations:** ^1^ School of Public Health, Guangdong Pharmaceutical University, Guangzhou, China; ^2^ The Prevention and Health Care Department, Shenzhen Maternity and Child Healthcare Hospital, Shenzhen, China; ^3^ Department of Health Management, Dongguan Qingxi Hospital, Qingxi Town, Dongguan, China; ^4^ Radiological Department, Guangzhou Panyu Central Hospital, Guangzhou, China; ^5^ The Health Care Office of the Hospital Infection Department, Affiliated Jiangmen Hospital of Sun Yat-sen University, Jiangmen, China; ^6^ The Affiliated Houjie Hospital, Guangdong Medical University, Dongguan, China; ^7^ Guangdong Provincial Institute of Public Health, Guangdong Provincial Center for Disease Control and Prevention, Guangzhou, China; ^8^ Precision Medicine Center, Dongguan Songshan Lake Central Hospital, Dongguan, China; ^9^ Department of Public Health and Preventive Medicine, School of Medicine, Jinan University, Guangzhou, China

**Keywords:** COVID-19, lockdown, birth weight, China, pregnant woman

## Abstract

**Background:**

During the special period of the global spread of COVID-19, pregnant women are sensitive groups to the impacts of COVID-19 epidemic. However, the effects of lockdown measures implemented in response to the COVID-19 on fetal birthweight remain unclear.

**Objectives:**

This study investigated the associations of COVID-19 lockdown with birth weight in Chinese population.

**Methods:**

We collected 730,153 data of participants from hospitals of five cities in the south of China, we defined the time period of level I response (1/23-2/24/2020) as level I lockdown, and women who were pregnant during level I lockdown as the exposure group. Women who were pregnant during the same calendar month from 2015 to 2019 were defined as the unexposed group. We quantitatively estimate the individual cumulative exposure dose by giving different weights to days with different emergency response levels. Generalized linear regression models were used to estimate the association between COVID-19 lockdown exposure with birth weight and risk of low birth weight (<2,500 g) and macrosomia (>4,000 g).

**Results:**

The birth weight of the exposed group is heavier than the unexposed group (3,238.52 vs. 3,224.11 g: adjusted *β* = 24.39 g [95% CI: 21.88, 26.91 g]). The exposed group had a higher risk of macrosomia (2.8% vs. 2.6%; adjusted OR = 1.17 [95% CI: 1.12, 1.22]). More obvious associations were found between COVID-19 lockdown and macrosomia in women who experienced the lockdown in their early pregnancy. Women who experienced the lockdown at their 4–7 weeks of pregnancy showed statistically significant heavier birth weight than unexposed group (after adjustment): *β* = 1.28 (95% CI: 1.11, 1.46) g. We also observed a positive association between cumulative exposure dose of COVID-19 lockdown in all pregnant women and birth weight, after divided into four groups, *Q*1: *β* = 32.95 (95% CI: 28.16, 37.75) g; *Q*2: *β* = 18.88 (95% CI: 14.12, 23.64) g; *Q*3: *β* = 19.50 (95% CI: 14.73, 24.28) g; *Q*4: *β* = 21.82 (95% CI: 17.08, 26.56) g. However, there was no statistically significant difference in the risk of low birth weight between exposed and unexposed groups.

**Conclusions:**

The COVID-19 lockdown measures were associated with a heavier birth weight and a higher risk of macrosomia. Early pregnancy periods may be a more susceptible exposure window for a heavier birth weight and a higher risk of macrosomia. We also observed a positive association between cumulative exposure dose of COVID-19 lockdown and birth weight. The government and health institutions should pay attention to the long-term health of the infants born during the COVID-19 lockdown period, and follow up these mothers and infants is necessary.

## 1 Introduction

Since the early of 2020, the COVID-19 was reported in more than 200 countries and regions, and has become a global pandemic. As of 17 December 2023, over 772 million confirmed cases and nearly seven million deaths have been reported globally (1).The health of global population has been severely affected. During the special period of the global spread of COVID-19, pregnant women and newborns are more vulnerable as sensitive groups. On the one hand, pregnant women's concerns and fears about the epidemic have increased their psychological burden. On the other hand, during the epidemic, insufficient hospital resources have resulted in pregnant women and newborns not receiving timely medical assistance. Insufficient food and social distancing prevent pregnant women from receiving sufficient care, leading to a series of health consequences, including preterm birth, miscarriage, and fetal growth restriction (FGR).

At present, the impact of COVID-19 on newborn birth weight has not been determined. An Ireland study reported an unprecedented decrease in the number of very low birthweight (VLBW) and extremely low birthweight (ELBW) in infants born in Ireland (2). And an increase in infants' birth weight was observed in Wuhan during the COVID-19 lockdown (3). However, other studies report that there is no negative impact of COVID-19 lockdown on birthweight (4, 5).

The above researches have demonstrated possible associations between COVID-19 lockdown and birth weight, but there are still several overlooked questions. First, as the severity of the epidemic changes, COVID-19 lockdown measures are being adjusted in most of countries and regions. However, all of these previous studies did not classify the exposure (2–4), which may be underestimate the impact of COVID-19 lockdown on adverse pregnancy outcomes. Second, previous studies have shown that the birth season of newborns is associated with their birth weight (6–8), therefore the impact of different seasons should be considered in the study of associations of COVID-19 lockdown with birth weight. Third, the follow-up time in in previous studies was too short to produce an outcome. Lastly, previous studies did not consider the pregnant woman's stage of pregnancy. Studies have shown that there are significant differences in the susceptibility of pregnant women to environmental factors during different pregnancy periods (9, 10).

To address these research gaps, we calculated the cumulative exposure separately according to the lockdown period of different intensities to avoid errors caused by exposure levels, considered the effects of seasonality and extend the cohort long enough to produce an outcome, analyzed the association of the COVID-19 lockdown on birthweight in South China. This study has positive significance for the health of pregnant women and newborns during the COVID-19 lockdown.

## 2 Methods

### 2.1 Study settings and subjects

**Figure 1:**
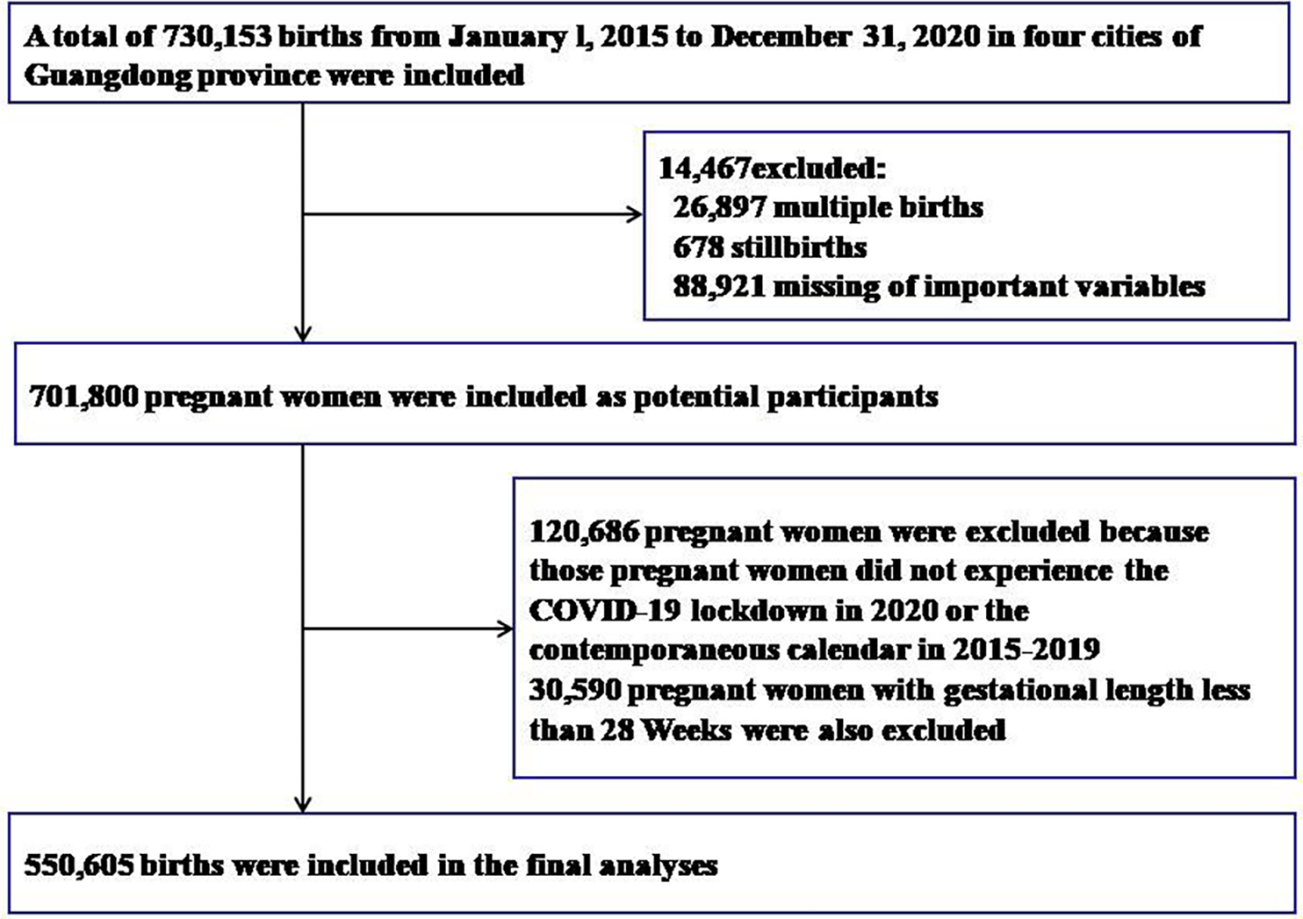
Selection process of study subjects.

We collected birth records of participants from hospitals of five cities in the south of China, including all hospitals in Foshan (*n* = 62), several hospitals in Dongguan (*n* = 2), Guangzhou (*n* = 1), Shenzhen (*n* = 1) and Jiangmen (*n* = 1). We included all birth data for all hospitals between February 1, 2015 and December 31, 2020 (*N* = 730,153). For this analysis, we excluding birth data with multiple births (*n* = 26,897), stillbirths (*n* = 678), or missing information on key variables (*n* = 88,921). In addition, 120,686 data were also excluded from this analysis because their pregnancies did not overlap with the COVID-19 lockdown in 2020 or the same calendar month from 2015 to 2019. Birth data with gestational length less than 28 weeks were also excluded (3) (*n* = 30,509). Overall, a sample size of 550,605 mother-infant pairs was included in the study (Figure 1). All the women were negative for the Novel Coronavirus test.

This study was approved by the Ethics Committee of Guangdong Center for Disease Control and Prevention (No. W96-027E-2020004).

### 2.2 Data collection

Each birth data contains the following information: Infant sex, date of birth, type of delivery (vaginal or cesarean), gestational weeks (GW), number of births, parity and major adverse pregnancy outcomes such as miscarriage and stillbirth. Above information about participants' health was obtained throughout Hospital information system or birth record system. We check the quality of the source data, implausible values and outliers were either corrected or recorded as missing.

### 2.3 Exposure assessment

**Figure 2:**
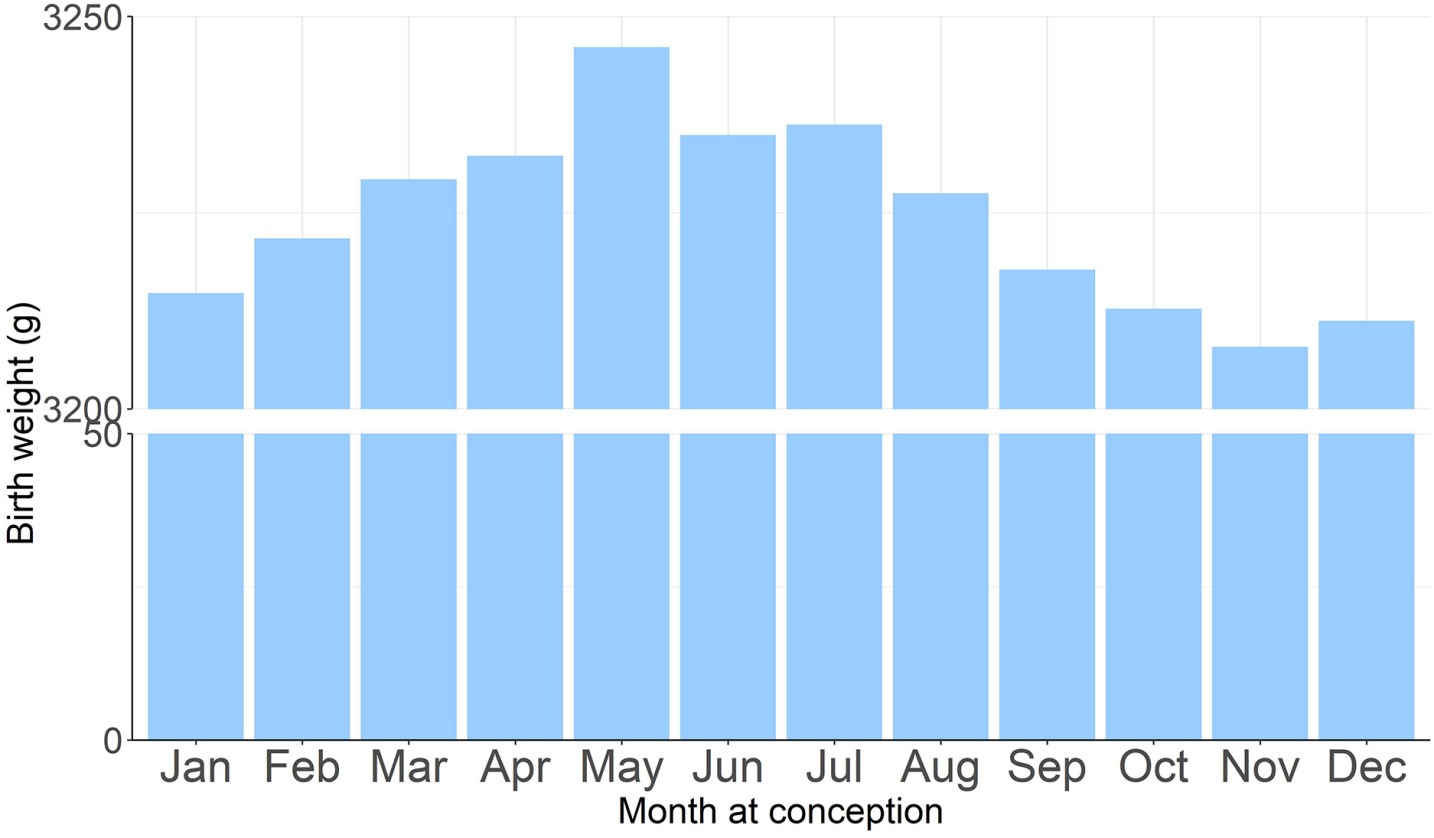
Birth weight in each calendar month during 2015–2019 (before the COVID-19 pandemic).

The National Emergency Response Plan for Public Emergencies by the China State Council defined 4 levels of emergency response: Level I (extremely serious), Level II (serious), Level III (relatively serious), and Level IV (common) (11). On January 23, the Guangdong Provincial Government took the lead in China in launching Level I response. The emergency response level was upgraded to level II on February 24 and upgraded to level III On May 9.We defined the time period of level I response (1/23-2/24/2020) as level I lockdown, and women who were pregnant during level I lockdown as the exposure group (*N* = 96,550). Our data showed significant differences in birth weight between the calendar months of pregnancy (Figure 2). To decrease the influence of seasonal effects, we defined women who became pregnant during the same calendar month from 2015 to 2019 as the unexposed group (*N* = 452,756).

Pregnant women and fetuses may have different sensitivities to COVID-19 lockdown at different stages of pregnancy. In order to explore the susceptible exposure window, they were further divided into 11 subgroups based on gestational age at 23 January 2020. The date of conception is calculated from gestational age and birth date. For example, women whose date of conception fell within the period of lockdown I were defined as Group I, while women whose gestational week was less than four weeks on 23 January 2020 were defined as Group II. Pregnant women whose GW less than 41 weeks were recorded as 41 weeks. The unexposed group was divided into 11 subgroups corresponding to the exposed group. For each pair of subgroups (exposed and unexposed), we evaluated the association between COVID-19 lockdown exposure and birth weight.

**Figure 3:**
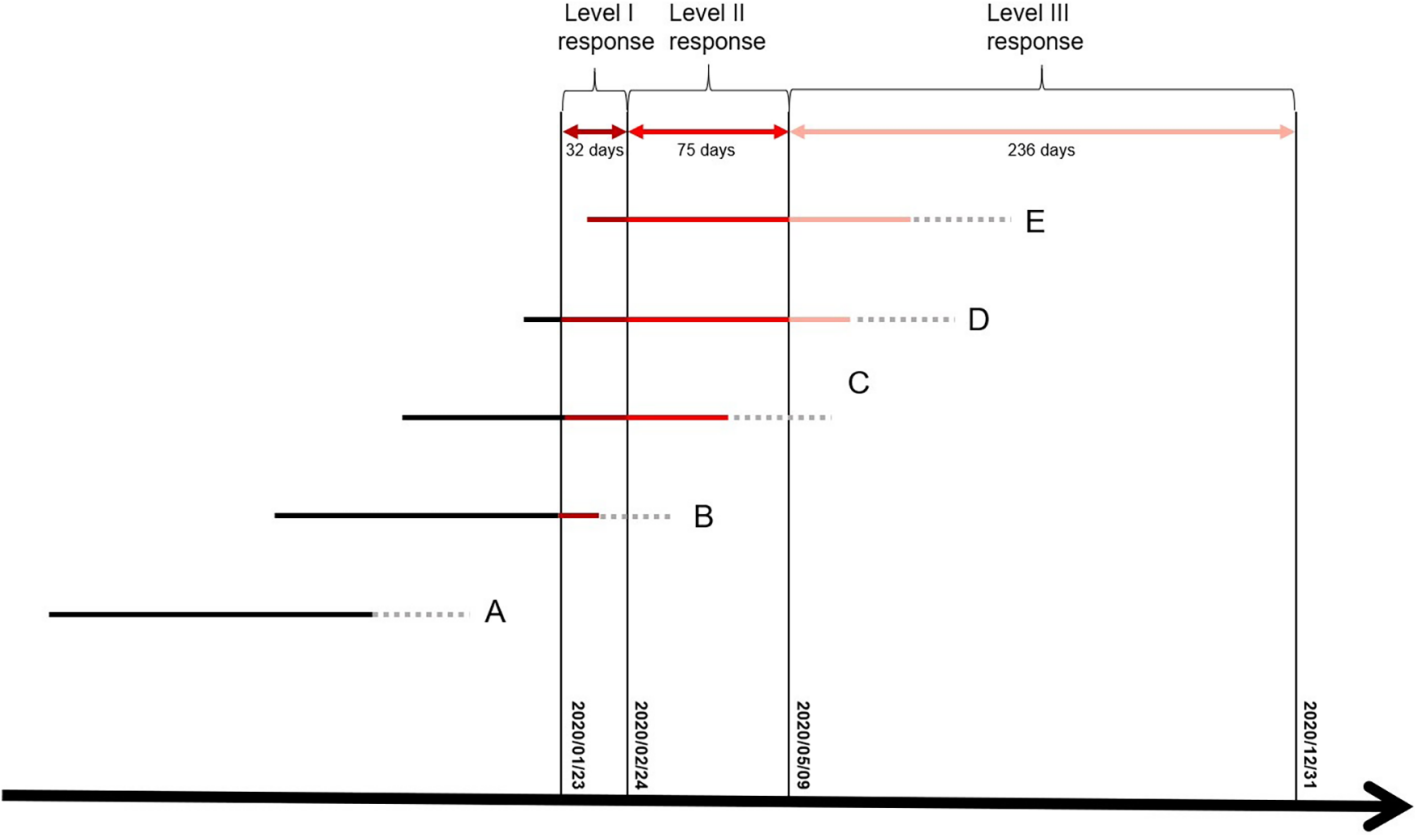
Approach to calculating individual cumulative exposure dose to lockdown in the first 37 GWs (12). 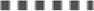: Weeks after 37 GWs. A, B, C, D and E represent subgroups of pregnant women with different GWs during the Level I lockdown; We assigned a weighting value of 3 to the days with Level I response, 2 to the days with Level II response, 1 to the days with Level III response, and 0 to days before lockdown (no exposure). Visit this link forcreative commons licence (Available at: https://bmcpregnancychildbirth.biomedcentral.com/articles/10.1186/s12884-021-04268-5#rightslink). No changes have been made to the referenced image.

The lockdown measures during level II and level III response periods are not as strong as those during the level I response period, so that the different effect of different lockdown measures level should be considered. Therefore, we quantitatively estimate the cumulative exposure dose of individuals by assigning different weights to days with different emergency response levels: 1/22/2020 or earlier was the no response period, 1/23-2/24/2020 was the level I response period, 2/25-5/9/2020 was the level II response period, and 5/10-12/31/2020 was the level III response period, with weights of 0, 3, 2, and 1, respectively (12). In addition, considering the potential effects of exposure time, we estimated only the cumulative exposure dose for the first 37 GW, based on the fact that the sample was reserved for full-term delivery only (Figure 3).

### 2.4 Outcome measures

Low birth weight (LBW) was defined as a birth weight of less than 2500 g (13). Normal birth weight was defined as birth weight between 2500 and 4,000 g. Macrosomia was defined as birth weight greater than 4,000 g (14).

### 2.5 Potential confounders

Considering biological rationality and literature review, we chose potential confounders such as maternal age, gestational age, parity, birth order, residential district, type of delivery and infant sex.

### 2.6 Statistical analysis

We used the chi-square test and *t*-test to compare differences of social demographic and gestational characteristics between the exposed and unexposed groups. We used the generalized linear model (GLM) to estimate the association between COVID-19 lockdown exposure and birth weight (linear regression) with adjustment for the confounders. Taking normal birth weight as reference, birth weight was further divided into low birth weight and macrosomia. We used multiple logistic regression model and we used birth weight as a classification variable in model. Similarly, GLM and multiple logistic regression models were used to estimate the association between cumulative exposure dose and birth weight. The cumulative exposure dose of the exposed group was divided into four quartiles (*Q*1, *Q*2, *Q*3 and *Q*4) to estimate the associations of each quartile of cumulative exposure (relative to unexposed) with birth weight. We did all of the analyses with R3.6.1. All tests were bilateral, and *P *< 0.05 was considered statistically significant.

## 3 Results

### 3.1 General characteristics of study participants

A comparison of general characteristics between the exposed group and unexposed group is shown in Table 1. The study included 549,306 women—96,550 in the exposed group and 452,756 in the unexposed group (Table 1). There were no significant differences in infant sex between exposed group and unexposed group. The unexposed group had higher proportions of participants over the age of 30, macrosomia, multiparity, and had lower proportions of participants with natural delivery than the exposed group.

**Table 1:** General characteristics of study participants.

	Unexposed group (*n* = 454,055) No. of participants (%)	Exposed group (*n* = 96,550) No. of participants (%)	*χ* ^2^	*P*
Maternal age (years)				
<24	46,101 (10.2)	7,986 (8.2)	595.14	<0.001
24–26	74,448 (16.4)	15,143 (15.7)		
27–29	108,738 (24.0)	22,625 (23.4)		
30–32	94,704 (20.9)	22,435 (23.2)		
33–35	66,436 (14.7)	15,181 (15.7)		
>35	62,329 (13.8)	13,180 (13.7)		
Residential city				
Guangzhou	19,163 (4.2)	2,842 (2.9)	1,576.50	<0.001
Dongguan	32,818 (7.2)	5,352 (5.5)		
Jiangmen	16,760 (3.7)	3,041 (3.1)		
Shenzhen	71,038 (15.7)	12,491 (12.9)		
Foshan	312,977 (69.1)	72,824 (75.4)		
Infant sex				
Male	240,268 (53.1)	51,209 (53.0)	0.03	0.872
Female	212,488 (46.9)	45,341 (47.0)		
Birthweight				
Low birth weight	9,880 (2.2)	1,892 (2.0)	30.82	<0.001
Normal birth weight	431,046 (95.2)	91,917 (95.2)		
Macrosomia	11,830 (2.6)	2,741 (2.8)		
Parity				
0 (Primiparas)	180,637 (39.9)	40,436 (41.9)	754.03	<0.001
1 (Multiparas)	229,319 (50.6)	44,866 (46.5)		
2–4 (Multiparas)	42,800 (9.5)	11,248 (11.6)		
Delivery type				
Natural delivery	275,873 (60.9)	60,767 (63.0)	2,198.40	<0.001
Operative vaginal delivery	13,774 (3.0)	438 (0.5)		
Cesarean delivery	160,843 (35.5)	34,633 (35.9)		
Other	2,266 (0.5)	712 (0.7)		
	Mean ± SD	Mean ± SD	*t*	*P*
Maternal age (years)	29.80 ± 7.09	30.03 ± 4.92	−9.93	<0.001
Gestational length (week Mean ± SD)	38.96 ± 1.05	38.89 ± 1.04	21.18	<0.001
Birthweight (g ± SD)	3,224.11 ± 393.61	3,238.52 ± 388.72	−10.35	<0.001

### 3.2 Associations of COVID-19 lockdown exposure with birth weight

As shown in Table 2, the birth weight of the exposed group is heavier than unexposed group (3,238.52 ± 388.72 g vs. 3,224.11 ± 393.61 g). The Level I response was significantly associated with a 24.39 (95% CI: 21.88, 26.91) g increase in birth weight in the entire sample after adjusting for confounders. After dividing into 11 subgroups, the first 9 subgroups showed statistically significant heavier birth weight in the exposed group than in the unexposed group, after adjusting for confounders. As shown in Table 3, We can also find a positive correlation between cumulative exposure dose and birth weight after adjusting for confounders. Each 100 unit increase in cumulative exposure was associated with a 6.54 (95% CI: 5.66, 7.41) g increase in birth weight. After the exposure group was divided into four groups according to the cumulative exposure amount, four groups were all showed statistically significant heavier birth weight than unexposed group (after adjustment): *Q*1: *β *= 32.95 (95% CI: 28.16, 37.75) g; *Q*2: *β* = 18.88 (95% CI: 14.12, 23.64) g; *Q*3: *β *= 19.50 (95% CI: 14.73, 24.28) g; *Q*4: *β *= 21.82 (95% CI: 17.08, 26.56) g.

**Table 2:** Associations of exposure to the COVID-19 lockdown with birth weight.

	No. of participants		Birth weight (g, Mean ± SD)		Mean difference in birth weight	
	Unexposed group	Exposed group^a^	Unexposed group	Exposed group^a^	Crude *β* (95% CI)	Adjusted *β* (95% CI)^b^
Gestational week at the beginning of the Level I lockdown						
All	452,756	96,550	3,224.11 ± 393.61	3,238.52 ± 388.72	14.41 (11.68, 17.14)	24.39 (21.88, 26.91)
Conception during the lockdown	60,095	10,685	3,217.94 ± 394.90	3,231.01 ± 387.33	13.07 (4.97, 21.17)	24.36 (16.90,31.83)
Prior to 4th	49,467	10,499	3,212.96 ± 395.86	3,234.47 ± 386.41	21.51 (13.21, 29.81)	31.01 (23.38, 38.64)
4th-7th	46,907	9,853	3,210.82 ± 393.69	3,235.31 ± 390.98	24.48 (15.94, 33.02)	37.34 (29.45, 45.22)
8th-11th	44,003	9,582	3,208.20 ± 390.46	3,229.51 ± 391.78	21.31 (12.68, 29.94)	33.48 (25.52, 41.046)
12th-15th	41,004	9,232	3,213.10 ± 392.77	3,230.23 ± 380.57	17.13 (8.31, 25.95)	29.77 (21.65, 37.90)
16th-19th	41,125	8,960	3,213.42 ± 390.74	3,224.62 ± 388.79	11.20 (2.28, 20.12)	22.04 (13.82, 30.26)
20th-23rd	37,313	8,288	3,227.75 ± 390.61	3,236.67 ± 385.65	8.91 (−0.36, 18.19)	20.87 (12.32, 29.43)
24th-27th	36,759	8,292	3,231.45 ± 392.04	3,246.64 ± 385.88	15.19 (5.87, 24.50)	22.33 (13.76, 30.90)
28th-31st	35,166	7,658	3,234.89 ± 391.58	3,240.79 ± 392.10	5.90 (−3.78, 15.58)	17.42 (8.49, 26.34)
32nd-36th	44,706	9,861	3,244.56 ± 395.52	3,246.40 ± 394.96	1.84 (−6.78, 10.46)	4.75 (−3.25, 12.75)
37th-41st	16,211	3,640	3,312.74 ± 394.32	3,319.40 ± 384.99	6.66 (−7.46, 20.77)	9.60 (−3.71, 22.91)

In calculating the cumulative exposure dose to lockdown, we assigned a weighting of 3 to days with Level I response, 2 to days with Level II response, 1 to days with Level III response, and 0 to other days.

^a^ Pregnant women who have experienced the COVID-19 lockdown (from 1/23/2020 to 2/24/2020) during any period of their pregnancy were defined as the exposed group. We further divided the exposed group into subgroups according to their gestational weeks (GW) on 1/23/2020, the beginning of lockdown.

^b^ Used the generalized linear model (GLM), adjusted for maternal age, parity, residential city, delivery type, gestational length and infant sex.

**Table 3:** Associations of cumulative exposure dose to the COVID-19 lockdown with birth weight.

	Exposure dose (Mean ± SD)		Birth weight (g, Mean ± SD)		Mean difference in birth weight (g)	
	Unexposed group	Exposed group	Unexposed group	Exposed group	Crude *β* (95% CI)	Adjusted *β* (95% CI)^a^
Cumulative exposure dosein the first 37 weeks during the Level I to the Level III lockdown^b^						
Per 100 unit increase in all participants	0 ± 0	268.31 ± 105.99	3,224.87 ± 393.63	3,235.35 ± 388.53	2.57 (1.62, 3.52)	6.54 (5.66, 7.41)
Categories of cumulative exposure dose						
Unexposed group	0 ± 0	–	3,224.87 ± 393.63	–	Reference	Reference
*Q*_1_ (<198)	–	109.38 ± 55.50	–	3,244.21 ± 392.24	19.32 (14.12, 24.52)	32.95 (28.16, 37.75)
*Q*_2_ (198–297)	–	255.88 ± 27.46	–	3,233.43 ± 385.89	8.73 (3.56, 13.90)	18.88 (14.12, 23.64)
*Q*_3_ (298–355)	–	328.45 ± 16.94	–	3,230.39 ± 387.94	5.52 (0.34, 10.71)	19.50 (14.73, 24.28)
*Q*_4_ (≥356)	–	376.91 ± 12.65	–	3,233.32 ± 387.86	8.45 (3.30, 13.60)^a^	21.82 (17.08, 26.56)
*P* for trend test	–	–	–	–	–	<0.001

^a^ Adjusted for maternal age, parity, residential city, delivery type, gestational length and infant sex.

^b^ The exposed group refers to the pregnant women who have experienced the COVID-19 lockdown in their first 37 GWs. The other participants were defined as the unexposed group. The individual cumulative exposure dose was calculated by combining the weightings with the overlap between their pregnancy period (≤37 GWs) and the three levels of responses. *Q*1–*Q*4 were defined as the cumulative exposure dose of the exposed group classified by quartiles, and the unexposed group was used as reference.

### 3.3 Associations of COVID-19 lockdown exposure with risk of LBW and macrosomia

Table 4 shows the comparison of LBW rate and macrosomia rate between exposed and unexposed groups. Compared to the unexposed group, exposed group had a higher macrosomia rate (2.8% vs. 2.6%) in the total sample. However, there was no statistically significant difference in the risk of low birth weight between the two groups. After adjusting for confounders, the risk of macrosomia in the exposed group was statistically significantly higher than that in the unexposed group, OR = 1.17 (95% CI: 1.12, 1.22). After dividing into 11 subgroups, all of the risk of macrosomia in the exposed group was higher than that unexposed group, and the first four subgroups, subgroup 6, subgroup 8 and subgroup 9 were statistically significant. More obvious associations were found between COVID-19 lockdown and macrosomia in women who experienced the lockdown in their early pregnancy. Women who experienced the lockdown at their 4–7 weeks of pregnancy showed statistically significant heavier birth weight than unexposed group (after adjustment): *β *= 1.28 (95% CI: 1.11, 1.46) g. As shown in Table 5, We can also find a positive correlation between cumulative exposure dose to COVID-19 lockdown and the risk of macrosomia after adjusting for confounders. Each 100 unit increase in the COVID-19 lockdown exposure during the first 37 GWs was significantly associated with 1.04 (95% CI: 1.02, 1.06) times higher risks in macrosomia. The adjusted ORs of macrosomia for the *Q*1, *Q*2, *Q*3 and *Q*4 quartiles of cumulative exposure (vs. no exposure) were 1.31 (1.21, 1.41), 1.10 (1.02, 1.20), 1.08 (0.99, 1.17), and 1.16 (1.07, 1.26), respectively. However, there was no statistically significant difference in the risk of low birth weight between exposed and unexposed groups.

**Table 4:** Associations of exposure to the COVID-19 lockdown with low birth weight andmacrosomia.

	Unexposed group (*n*, %)			Exposed group (*n*, %)^a^			OR forlow birth weight and macrosomia (95% CI)			
	Low birth weight	Normal birth weight	Macrosomia	Low birth weight	Normal birth weight	Macrosomia	Low birth weight		Macrosomia	
							Crude OR	Adjusted OR^b^	Crude OR	Adjusted OR^b^
Gestational week at the beginning of the LevelI lockdown										
All	9,880 (2.2)	431,046 (95.2)	11,830 (2.6)	1,892 (2.0)	91,917 (95.2)	2,741 (2.8)	0.90 (0.85, 0.94)	0.85 (0.81, 0.89)	1.09 (1.04, 1.13)	1.17 (1.12, 1.22)
Conception during the lockdown	1,359 (2.26)	57,226 (95.2)	1,510 (2.5)	220 (2.1)	10,153 (95.0)	312 (2.9)	0.91 (0.79, 1.05)	0.86 (0.74, 1.00)	1.16 (1.03, 1.32)	1.27 (1.12, 1.44)
Prior to 4th	1,165 (2.4)	47,095 (95.2)	1,207 (2.4)	192 (1.8)	10,029 (95.5)	278 (2.6)	0.77 (0.66, 0.90)	0.73 (0.62, 0.85)	1.08 (0.95, 1.23)	1.17 (1.03, 1.34)
4th–7th	1,042 (2.2)	44,744 (95.4)	1,121 (2.4)	203 (2.1)	9,377 (95.2)	273 (2.8)	0.93 (0.80, 1.08)	0.86 (0.73, 0.99)	1.16 (1.02, 1.33)	1.28 (1.11, 1.46)
8th–11th	1,015 (2.3)	41,975 (95.4)	1,013 (2.3)	209 (2.1)	9,131 (95.4)	242 (2.5)	1.95 (0.81, 1.10)	0.86 (0.74, 1.01)	1.10 (0.95, 1.27)	1.19 (1.03, 1.37)
12th–15th	967 (2.4)	39,040 (95.3)	967 (2.4)	208 (2.3)	8,515 (95.0)	237 (2.6)	0.80 (0.68, 0.94)	0.73 (0.62, 0.86)	1.02 (0.88, 1.17)	1.08 (0.93, 1.25)
16th–19th	980 (2.3)	39,178 (95.3)	1,025 (2.4)	208 (2.3)	8,520 (95.0)	238 (2.7)	0.97 (0.84, 1.13)	0.92 (0.79, 1.07)	1.13 (0.97, 1.30)	1.23 (1.07, 1.43)
20th–23rd	806 (2.2)	35,562 (95.3)	945 (2.5)	170 (2.1)	7,902 (95.3)	216 (2.6)	0.95 (0.80, 1.12)	0.88 (0.74, 1.04)	1.03 (0.89, 1.19)	1.12 (0.96, 1.30)
24th–27th	788 (2.1)	34,942 (95.1)	1,029 (2.8)	150 (1.8)	7,881 (95.0)	261 (3.1)	0.84 (0.71, 1.01)	0.82 (0.69, 0.99)	1.12 (0.98, 1.29)	1.19 (1.04, 1.38)
28th–31st	714 (2.0)	33,464 (95.2)	988 (2.8)	142 (1.9)	7,282 (95.1)	234 (3.1)	0.91 (0.76, 1.10)	0.86 (0.71, 1.03)	1.09 (0.94, 1.26)	1.20 (1.03, 1.39)
32nd–36th	881 (2.0)	42,427 (94.9)	1,398 (3.1)	188 (1.9)	9,362 (94.96)	311 (3.2)	0.97 (0.82 1.13)	1.01 (0.85, 1.18)	1.01 (0.89, 1.14)	1.05 (0.92, 1.19)
37nd–41th	163 (1.0)	15,393 (95.0)	655 (4.0)	35 (1.0)	3,457 (95.0)	148 (4.1)	0.96 (0.66, 1.38)	1.01 (0.69, 1.46)	1.01 (0.84, 1.21)	1.04 (0.86, 1.25)

^a^ Pregnant women who have experienced the COVID-19 lockdown (from 1/23/2,020 to 2/24/2020) during any period of their pregnancy were defined as the exposed group. We further divided the exposed group into subgroups. according to their gestational weeks (GW) on 1/23/2020, the beginning of lockdown.

^b^ Adjusted for maternal age, parity, residential city, delivery type, gestational length and infant sex.

**Table 5:** Associations of cumulative exposure dose to the COVID-19 lockdown with low birth weight and macrosomia.

	Exposure dose in unexposed group (Mean ± SD)	Exposure dose in exposed group (Mean ± SD)			OR for low birth weight and macrosomia (95% CI)			
		Low birth weight	Normal birth weight	Macrosomia	Low birth weight		Macrosomia	
					Crude OR	Adjusted OR^a^	Crude OR	Adjusted OR^a^
Cumulative exposure dosein the first 37 weeks during Level I to Level 3 lockdown^b^								
Per 100 unit increase	0 ± 0	270.63 ± 103.99	268.43 ± 105.94	262.51 ± 108.84	0.98 (0.96, 0.99)	0.95 (0.94, 0.97)	1.01 (0.99 1.03)	1.04 (1.02, 1.06)
Categories of cumulative exposure dose								
Unexposed group	0 ± 0	–	–	–	Reference	Reference	Reference	Reference
*Q*_1_ (<198)	–	109.56 ± 54.50	109.34 ± 55.52	110.41 ± 55.47	0.88 (0.80, 0.97)	0.80 (0.73, 0.92)	1.20 (1.11, 1.29)	1.31 (1.21, 1.41)
*Q*_2_ (198–296)	–	257.14 ± 25.87	255.86 ± 27.48	255.74 ± 27.88	0.93 (0.85, 1.02)	0.88 (0.80, 0.99)	1.02 (0.94, 1.11)	1.10 (1.02, 1.20)
*Q*_3_ (297–354)	–	328.77 ± 16.62	328.42 ± 16.94	329.38 ± 17.14	0.97 (0.88, 1.06)	0.89 (0.81 1.01)	0.98 (0.91, 1.07)	1.08 (0.99, 1.17)
*Q*_4_ (≥355)	–	376.38 ± 13.01	376.95 ± 12.64	376.04 ± 12.49	0.90 (0.82, 0.99)	0.83 (0.76, 1.03)	1.05 (0.97, 1.14)	1.16 (1.07, 1.26)
*P* for trend test	–	–	–	–	–	0.55	–	<0.05

^a^ Adjusted for maternal age, parity, residential city, delivery type, gestational length and infant sex.

^b^ The exposed group refers to the pregnant women who have experienced the COVID-19 lockdown in their first 37 GWs. The rest of included participants were defined as the unexposed group. The individual cumulative exposure dose was calculated by combining the weightings with the overlap between their pregnancy period ≤37 GWs and the three levels of responses. *Q*_1_–*Q*_4_ were defined as the cumulative exposure dose of the exposed group classified by quartiles, and the unexposed group were used as reference.

## 4 Discussion

Our study of the association of COVID-19 lockdown measures and birth weight showed that COVID-19 lockdown was associated with a higher birth weight and a higher risk of macrosomia. Early and middle pregnancy might be the susceptible exposure window of the effect of COVID-19 lockdown on birth weight and macrosomia. However, no association between COVID-19 locking measures and LBW risk was observed.

There are several possible reasons for the increase in birth weight and risk of macrosomia during COVID-19 lockdown: First, dietary and nutritional status and physical activity of the pregnant women during COVID-19 lockdown period may be associated with increased weight in pregnant women, which may lead to increased birth weight in newborns (15, 16). On the one hand, during the COVID-19 lockdown, especially at the beginning of the lockdown, people stocked up on nonperishable food because of lack of supplies and panic mood. Compared with the usual, the intake of carbohydrate is higher, the intake of vitamins and dietary fiber is less, and the distribution of nutrients is very uneven (3). It can result in higher weight and a higher BMI in pregnant women. On the other hand, because of lockdown measures during the outbreak, most people were forced to work at home, and some even be unemployed. Less outdoor time and less exercise increase the risk of obesity (17). The above two causes of maternal weight increase may be the reason for the infant's birth weight increase. Secondly, pregnant women during the lockdown were facing heavier psychological pressure, including family relationships and economic issue (18). Studies have shown that pregnant women who experience high levels of psychological stress during pregnancy have an increased risk of macrosomia (19). Previous research showed that brain-derived neurotrophic factor (BDNF) are neutrophils, which are thought to play an important role in the growth and development of the placenta and fetus. One possible hypothesis to explain the relationship between maternal stress and birth weight is that changes in intrauterine concentrations of BDNF are associated with maternal stress and fetal growth and development (19). Lastly, it may also because pregnant women were unwilling or unable to go out for maternity check-ups during the epidemic, abnormal fetal weight cannot be detected in time. Our previous research also found that COVID-19lockdown increases the risk of Gestational diabetes, and Gestational diabetes is an important risk factor for macrosomia (20).

We further found that women in the early stages of pregnancy during Level I lockdown had a heavier newborn birth weight and higher risk of macrosomia. The effect of COVID-19 lockdown is more significant in the early stage of pregnancy. It may be explained that the early stage of pregnancy is a critical time for fetal development. Previous studies have suggested that the early stages of pregnancy may be a susceptible exposure window of association of fine particulate matter and risk of preterm birth (9, 10). The early pregnancy is also a critical period for the occurrence of Gestational diabetes, and Gestational diabetes is related to the increased risk of macrosomia. Gestational diabetes leads to increased insulin resistance in pregnant women, and blood sugar enters the fetal circulation through the placenta. The excess glucose in the fetal body is converted into body fat, leading to macrosomia (21). It could also be explained that women in the early stages of pregnancy during lockdown are more likely to have longer lockdown period and higher lockdown cumulative exposure, which is more likely to observe the outcome.

Our results are similar to some previous studies: some studies have shown that COVID-19 lockdown is related to increased birth weight (2, 3, 22, 23). However, our results are not completely consistent with the results of some other studies. In some studies, no statistically significant changes in birth weight were found before and after COVID-19 lockdown (4, 5). Possible reasons are as follows: 1. Studies in different regions have different economic and social backgrounds, and standards and measures for COVID-19 lockdown vary from country to country. These complex factors may lead to different research results. 2. The sample size was insufficient to explore the association between lockdown exposure and birth weight. 3. COVID-19 lockdown effects on health are likely to continue for several months. The time of observation varies in different studies. Studies with shorter observation periods may not be sufficient to produce outcomes. 4. Most previous studies did not take seasonal effects and pregnancy stages into account, which may lead to an underestimate of association between COVID-19 lockdown and birth weight.

There are some advantages in our study. First, we quantitatively evaluated the COVID-19 lockdown exposure and analyzed the exposure-response association between COVID-19 lockdown and birth weight. Second, our study has sufficient sample size and a long time span of research data. It is beneficial for us to conduct subgroup analysis and explore the susceptible exposure window of COVID-19 lockdown on the impact of birth weight. Third, previous studies have shown that birth weight of newborns is influenced by the birth season. Therefore the impact of different seasons should be considered in the study of associations of COVID-19 lockdown with birth weight. We estimated the difference in birth weight between the exposed group and newborns born in the same calendar months from 2015 to 2019.

There are also some limitations in our study. First, appropriate physical activity during pregnancy has a positive impact on the health of both the mother and fetus, including a healthy birth weight. In fact, long-term studies have shown that women who exercise regularly during pregnancy are more likely to give birth to a suitable birth weight baby (24). However, weight, BMI and physical activity intensity information of the pregnant women were not available in the study, so we can't analyze the potential impact of these variables. Second, COVID-19 lockdown measures vary greatly among different countries and regions, and their impact on birth weight may have difference. The study was conducted in only a few cities in Guangdong Province and the sample size was uneven between cities which limit the extrapolation of our results. Third, this study only selected fetal information from hospital information systems or birth record systems. However, some pregnancy losses may not be recorded in the hospital information system, which may bias the association between COVID-19 lockdown and birthweight.

As the COVID-19 lockdown may have a variety of unpredictable health impacts, it is important to disseminate relevant information on this issue for the long-term health on newborns. Parents who experience COVID-19 lockdown during pregnancy period should pay more attention to the growth and development of their infants. The public health departments should regularly follow up the newborns whose mother experienced COVID-19 lockdown during pregnancy, especially those who were overweight. At the same time, it is necessary to pay more attention to the mental health of postpartum women.

## 5 Conclusions

This study compared birth weights of newborns before and after the COVID-19 lockdown and showed that COVID-19 lockdown was associated with a higher birth weight and a higher risk of macrosomia. Early months of pregnancy might be the susceptible exposure window of COVID-19 lockdown. Nowadays, COVID-19 is no longer the primary health threat in people's life, but the long-term health of infants born during COVID-19 lockdown period is still well worth noticing and exploring. This study has certain significance for the public health policy and clinical treatment related to maternal and infant health in the later stage of COVID-19. The government and health institutions should pay attention to the long-term health of the infants born during the COVID-19 lockdown period, and follow up these mothers and infants is necessary.

## Data Availability

The raw data supporting the conclusions of this article will be made available by the authors, without undue reservation.
